# Impact of the COVID-19 pandemic on the selection of chest imaging modalities and reporting systems: a survey of Italian radiologists

**DOI:** 10.1007/s11547-021-01385-1

**Published:** 2021-07-01

**Authors:** Andrea Borghesi, Nicola Sverzellati, Roberta Polverosi, Maurizio Balbi, Elisa Baratella, Marco Busso, Lucio Calandriello, Giancarlo Cortese, Alessandra Farchione, Roberto Iezzi, Stefano Palmucci, Ilaria Pulzato, Cristiano Rampinelli, Chiara Romei, Adele Valentini, Roberto Grassi, Anna Rita Larici

**Affiliations:** 1 Department of Medical and Surgical Specialties, Radiological Sciences and Public Health, University of Brescia, ASST Spedali Civili of Brescia, Piazzale Spedali Civili, 1, 25123 Brescia, Italy; 2 Radiological Sciences, Department of Medicine and Surgery, University Hospital of Parma, Parma, Italy; 3 Antoniano Diagnostic Institute, Padova, Italy; 4 Department of Radiology, Cattinara Hospital, University of Trieste, Trieste, Italy; 5 Department of Radiology, Department of Oncology, San Luigi Gonzaga University Hospital, University of Turin, Turin, Italy; 6 Department of Diagnostic Imaging, Oncological Radiotherapy and Hematology, Fondazione Policlinico Universitario “A. Gemelli” IRCCS, Roma, Italy; 7 Department of Radiology, Maria Vittoria Hospital, ASL Città Di Torino, Turin, Italy; 8 Department of Radiological and Hematological Sciences, Section of Radiology, Università Cattolica del Sacro Cuore, Roma, Italy; 9 Department of Medical Surgical Sciences and Advanced Technologies G.F. Ingrassia- Radiology I Unit, University Hospital Policlinico G. Rodolico-San Marco, University of Catania, Catania, Italy; 10 Department of Radiology, San Martino Hospital, University of Genoa, Genoa, Italy; 11 Department of Medical Imaging and Radiation Sciences, IEO European Institute of Oncology IRCCS, Milan, Italy; 12 Department of Diagnostic and Imaging, University Hospital of Pisa, Pisa, Italy; 13 Department of Radiology, San Matteo Polyclinic Foundation IRCCS, Pavia, Italy; 14 Department of Precision Medicine, University of Campania “L. Vanvitelli”, Naples, Italy; 15 Italian Society of Medical and Interventional Radiology (SIRM), SIRM Foundation, Milan, Italy

**Keywords:** SARS-CoV-2, COVID-19, Diagnostic imaging, Surveys and questionnaires

## Abstract

**Purpose:**

Chest imaging modalities play a key role for the management of patient with coronavirus disease (COVID-19). Unfortunately, there is no consensus on the optimal chest imaging approach in the evaluation of patients with COVID-19 pneumonia, and radiology departments tend to use different approaches. Thus, the main objective of this survey was to assess how chest imaging modalities have been used during the different phases of the first COVID-19 wave in Italy, and which diagnostic technique and reporting system would have been preferred based on the experience gained during the pandemic.

**Material and Methods:**

The questionnaire of the survey consisted of 26 questions. The link to participate in the survey was sent to all members of the Italian Society of Medical and Interventional Radiology (SIRM).

**Results:**

The survey gathered responses from 716 SIRM members. The most notable result was that the most used and preferred chest imaging modality to assess/exclude/monitor COVID-19 pneumonia during the different phases of the first COVID-19 wave was computed tomography (51.8% to 77.1% of participants). Additionally, while the narrative report was the most used reporting system (55.6% of respondents), one-third of participants would have preferred to utilize structured reporting systems.

**Conclusion:**

This survey shows that the participants’ responses did not properly align with the imaging guidelines for managing COVID-19 that have been made by several scientific, including SIRM. Therefore, there is a need for continuing education to keep radiologists up to date and aware of the advantages and limitations of the chest imaging modalities and reporting systems.

## Introduction

One year after the emergence of the first Italian clusters of severe acute respiratory syndrome coronavirus 2 (SARS-CoV-2) infection, Italy is currently experiencing a third wave of the coronavirus pandemic. Reverse transcription-polymerase chain reaction (RT-PCR) testing remains the reference standard for the definitive diagnosis of SARS-CoV-2 infection; however, it is well known that the sensitivity of RT-PCR is not optimal [1–3]. Therefore, in patients displaying clinical features suggestive of coronavirus disease (COVID-19), a negative RT-PCR result cannot exclude the possibility of a SARS-CoV-2 infection. Thus, in this context, chest imaging modalities continue to play a crucial role in the evaluation of symptomatic patients with an intermediate-to-high pre-test probability of SARS-CoV-2 infection or in resource-constrained environments where rapid decision-making is paramount to ensure proper treatment [4]. Specifically, for symptomatic patients exhibiting clinical features suggestive of COVID-19 pneumonia, the World Health Organization suggests the use of chest imaging modalities in cases where RT-PCR testing is negative, or in situations where care providers must decide between hospitalization and discharge of a patient [5].

Chest computed tomography (CT), chest X-ray (CXR), and lung ultrasonography (LUS) are the most commonly used imaging modalities for the management of patients with COVID-19 pneumonia [6–24].

Chest CT, especially high-resolution CT (HRCT), is the most sensitive imaging technique for the detection of lung abnormalities, and there is very good agreement among radiologists that chest CT is effective in confirming or excluding the possibility of a COVID-19 pneumonia [1–5, 13–17]. Additionally, chest CT is the most effective imaging modality in confirming or excluding thoracic complications in patients with COVID-19 pneumonia [21].

However, along with the issue related to radiation exposure, one of the main drawbacks of CT in COVID-19 diagnosis is its moderate to low specificity [13, 16]. Therefore, using CT as a first-line diagnostic tool to confirm or exclude the possibility of COVID-19 infection is not recommended by several scientific societies [15], including the Italian Society of Medical and Interventional Radiology (SIRM) [25].

Although the sensitivity of CXR for the detection of lung abnormalities is relatively low, especially in the early stages of COVID-19 pneumonia [4, 8, 13], there is ample evidence in the literature that the use of CXR can be advantageous in the management of patients with COVID-19 [6–13]. Therefore, CXR is generally used as the first-line imaging modality for evaluating and monitoring COVID-19 pneumonia, particularly in areas with a high number of infected individuals [6–13, 15, 25].

LUS is another valid diagnostic tool with a high sensitivity for the detection and monitoring of lung abnormalities in patients with COVID-19 pneumonia [22–24]. In suspected or confirmed cases, LUS is a rapid, radiation-free, bedside diagnostic tool that can be helpful in the management of patients [22–24]. However, the main drawbacks of LUS include its low specificity and the fact that it can only evaluate the peripheral-subpleural regions of the lung parenchyma [23]. Additionally, the utility of LUS as a diagnostic tool is highly operator-dependent, and there are no clear guidelines on its use in the management of patients with COVID-19 [4, 23]. In combination with CXR, however, LUS could be used during triage or in intensive care units to improve decision-making and to reduce the need for CT examinations, minimizing the risk of cross infection associated with the transport of COVID-19 patients.

Unfortunately, there is currently no consensus on the optimal chest imaging approach in the evaluation of patients with clinically suspected COVID-19 pneumonia, and even though recommendations do exist [4, 5, 15, 25, 26], radiology departments in Italy tend to use different approaches in the evaluation of such cases. Thus, we decided to conduct a survey among the Italian radiologists, members of the SIRM, to assess how chest imaging modalities have been used during the different phases of the first wave of COVID-19 and which diagnostic technique would have been preferred based on the experience gained during the pandemic. In addition, we also investigated which reporting systems for chest imaging findings in COVID-19 pneumonia were used and which method would have been preferred during the first wave of COVID-19.

## Materials and methods

### Study design

This national survey was promoted by the Board of the College of Chest Radiologists of the SIRM to investigate the impact of the COVID-19 pandemic on the selection of chest imaging modalities and reporting systems in patients with suspected or confirmed COVID-19 pneumonia. Institutional Review Board approval was not required for this study because of its survey nature.

The first draft of the questionnaire was prepared by two chest radiologists (A.B. and A.R.L.) at the beginning of May 2020, which coincided with the end of the first COVID-19 lockdown in Italy. The draft was shared with all members of the Board of the College of Chest Radiologists of the SIRM to assess the need for any changes or additions to the survey. The final version of the survey, which consisted of 26 questions (25 single-choice and one multiple-choice), was approved by all members of the Board and by the President of the SIRM (R.G.) on May 15, 2020.

SurveyMonkey, an online survey software, was used to create an electronic version of the questionnaire. On May 25, 2020, an e-mail containing the link to participate in the survey was sent to all members of the SIRM. To increase the number of participants, a reminder email was sent to all members on June 3, 2020. The survey link was generated so that each respondent was required to answer all questions in a single session. The survey questionnaire was prepared to collect baseline and specific information regarding the characteristics of the participants and their diagnostic approach to evaluating patients in different phases (*phase 1, phase 2, and monitoring*) of the first wave of the pandemic. Specifically, the first phase of the COVID-19 pandemic (beginning on February 21, 2020 and ending on May 3, 2020) corresponded to a period of high to moderate prevalence of SARS-CoV-2 infection, whereas the second phase of the COVID-19 pandemic (beginning on May 4, 2020) corresponded to a period of low prevalence of SARS-CoV-2 infection. The complete list of survey questions and their possible responses are presented in Table 1.

**Table 1:** Full list of questions and response options of the survey

Questions and response options	Replies (%)
Q1. How many years have you been working as a radiologist (including years of residency)?	
< 10 years	165 (23.0)
10 – 20 years	243 (33.9)
21 – 30 years	153 (21.4)
> 30 years	155 (21.7)
Q2. In which geographical area of Italy do you work?	
North	391 (54.6)
Center	182 (25.4)
South	101 (14.1)
Islands	42 (5.9)
Q3. What is your main workplace?	
University hospital	198 (27.7)
Nonuniversity public hospital	373 (52.1)
Accredited private hospital	102 (14.3)
Other private facility	43 (6.0)
Q4. What position do you hold at the facility where you work?	
Resident	56 (7.8)
Consultant physician	453 (63.3)
Physician manager	123 (17.2)
Private consultant	84 (11.7)
Q5. During the phase 1 of the COVID-19 emergency, how many patients with SARS-CoV-2 infection were admitted to the facility where you work?	
< 50 patients	217 (30.3)
50 – 500 patients	371 (51.8)
501 – 1500 patients	80 (11.2)
> 1500 patients	48 (6.7)
Q6. During the phase 1 of the emergency, which diagnostic method did you most use to assess/exclude COVID-19 pneumonia?	
CXR	305 (42.6)
CXR + LUS	23 (3.2)
HRCT	334 (46.7)
Conventional CT	54 (7.5)
Q7. During the phase 1 of the emergency, which diagnostic method did you use less frequently to assess/exclude COVID-19 pneumonia?	
CXR	88 (12.3)
CXR + LUS	431 (60.2)
HRCT	84 (11.7)
Conventional CT	113 (15.8)
Q8. Who performs LUS at the facility where you work? (*multiple choice*)	
Radiologist	183 (25.6)
Emergency physician	311 (43.4)
Pulmonologist/internist	285 (39.8)
Critical care physician	274 (38.3)
Q9. Based on the experience gained during phase 1, which diagnostic method would you have preferred to assess/exclude COVID-19 pneumonia?	
CXR	110 (15.4)
CXR + LUS	54 (7.5)
HRCT	464 (64.8)
Conventional CT	88 (12.3)
Q10. Since the beginning of the phase 2, which diagnostic method did you most use to assess/exclude COVID-19 pneumonia?	
CXR	232 (32.4)
CXR + LUS	14 (2.0)
HRCT	404 (56.4)
Conventional CT	66 (9.2)
Q11. Since the beginning of the phase 2, which diagnostic method did you use less frequently to assess/exclude COVID-19 pneumonia?	
CXR	109 (15.2)
CXR + LUS	408 (57.0)
HRCT	83 (11.6)
Conventional CT	116 (16.2)
Q12. For the phase 2, which diagnostic method would you prefer to assess/exclude COVID-19 pneumonia?	
CXR	129 (18.0)
CXR + LUS	77 (10.8)
HRCT	435 (60.8)
Conventional CT	75 (10.5)
Q13. During the COVID-19 emergency (both phase 1 and 2), which diagnostic method did you most use for monitoring COVID-19 pneumonia?	
CXR	324 (45.3)
CXR + LUS	21 (2.9)
HRCT	308 (43.0)
Conventional CT	63 (8.8)
Q14. Based on the experience gained during the COVID-19 emergency, which diagnostic method would you have preferred for monitoring COVID-19 pneumonia?	
CXR	232 (32.4)
CXR + LUS	63 (8.8)
HRCT	349 (48.7)
Conventional CT	72 (10.1)
Q15. During the COVID-19 emergency (both phase 1 and 2), which reporting format did you use in patients with COVID-19 pneumonia?	
Narrative report	398 (55.6)
Narrative report + visual score	234 (32.7)
Narrative report + quantitative score	22 (3.1)
Structured report + score	62 (8.7)
Q16. Based on the experience gained during the COVID-19 emergency, which reporting format would you have preferred in patients with COVID-19 pneumonia?	
Narrative report	129 (18.0)
Narrative report + visual score	186 (26.0)
Narrative report + quantitative score	140 (19.6)
Structured report + score	261 (36.5)
Q17. For CT images with lung window setting, which slice thickness did you use to assess/exclude COVID-19 pneumonia?	
≤ 1 mm	224 (31.3)
> 1 – 1.5 mm	385 (53.8)
> 1.5–2.5 mm	97 (13.6)
> 2.5 mm	10 (1.4)
Q18. For CT images with lung window setting, which reconstruction algorithm did you use to assess/exclude COVID-19 pneumonia?	
FBP with high spatial frequency	366 (51.1)
FBP with low spatial frequency	67 (9.4)
Iterative with high spatial frequency	248 (34.6)
Iterative with low spatial frequency	35 (4.9)
Q19. In chest CT, which of the following display modalities did you most use to assess the characteristics, extent and distribution of COVID-19 pneumonia?	
Axial images (only)	113 (15.8)
Axial, coronal, and sagittal MPR images	545 (76.1)
Interactive 3D CT viewer	46 (6.4)
Advanced 3D analysis software	12 (1.7)
Q20. During the phase 1 of the emergency, did you use low-dose chest CT protocol to assess/exclude COVID-19 pneumonia?	
Never	283 (39.5)
Only in young patients	242 (33.8)
Often, with the exception of obese patients	136 (19.0)
Always	55 (7.7)
Q21. Since the beginning of the phase 2, are you using low-dose chest CT protocol to assess/exclude COVID-19 pneumonia?	
Never	245 (34.2)
Only in young patients	243 (33.9)
Often, with the exception of obese patients	167 (23.3)
Always	61 (8.5)
Q22. In chest CT, did you use intravenous contrast media to evaluate patients with COVID-19 pneumonia?	
Never	164 (22.9)
Yes, but only in cases of suspected pulmonary embolism	491 (68.6)
Yes, but only when requested by clinicians	58 (8.1)
Always	3 (0.4)
Q23. In patients with COVID-19 and suspected pulmonary embolism, did you use DECT protocols with iodine maps?	
Never, we don’t have a DECT scanner	580 (81.0)
Rarely, even if we have a DECT scanner	80 (11.2)
Sometimes, we have not defined a dedicated protocol	31 (4.3)
Often, we have defined a dedicated protocol	25 (3.5)
Q24. During the phase 1 of the COVID-19 emergency, have staff meetings been organized at your department?	
Never	221 (30.9)
Rarely	186 (26.0)
Sometimes	206 (28.8)
Regularly (weekly)	103 (14.4)
Q25. Since the beginning of the phase 2 of the COVID-19 emergency, are staff meetings organized at your department?	
Never	263 (36.7)
Rarely	213 (29.8)
Sometimes	168 (23.5)
Regularly (weekly)	72 (10.0)
Q26. Hoping that pandemics such as COVID-19 will no longer occur, do you think conferences or courses dedicated to the imaging of pulmonary infections can be useful?	
Yes	344 (48.0)
Yes, in particular on viral infections and differential diagnoses	329 (46.0)
No, I have been sufficiently up to date on this topic	29 (4.0)
No, for a few years I don’t want to hear about pulmonary infection	14 (2.0)

Data are presented as numbers (%); CXR, chest X-ray; LUS, lung ultrasonography; HRCT, high-resolution computed tomography; CT, computed tomography; FBP, filtered back projection; MPR, multiplanar reformation; 3D, three-dimensional; DECT, dual-energy computed tomography

### Data analysis

The participants’ responses are presented as numbers and percentages. We conducted subanalyses to assess differences in participant responses based on the number of years each respondent had worked as a radiologist, their workplace, their geographical area of work, and the number of patients who had been hospitalized for SARS-CoV2 infection. We also examined whether and how the experience gained during the pandemic impacted the choice of imaging techniques and reporting systems used in different phases of the first wave of COVID-19.

To analyze differences in participant responses between the sub-groups, the Cochran-Armitage test for trend was used. The data were collected in an anonymous manner and analyzed in aggregate form using SurveyMonkey and a commercially available statistical analysis software package (MedCalc Statistical Software version 19.7, Ostend, Belgium). The cut-off value for statistical significance was set at *p* < 0.05.

## Results

The survey gathered responses from 716 SIRM members, corresponding to 6.8% of all members. A summary of all of the participants’ responses is provided in Table 1.

### Characteristics of the survey participants (Q1–Q5)

Most participants (77%) had radiological experience of at least 10 years, including the number of years in residency. More than half of the participants (52.1%) were employed in a nonuniversity public hospital, whereas approximately one-third (27.7%) were employed in university hospitals. While 453/716 (63.3%) of the respondents were consultant physicians (including university staff), only 56/716 (7.8%) were residents.

More than half of the participants (54.6%) worked in Northern Italy, about a quarter (25.4%) worked in Central Italy, and 20% of the respondents worked in Southern Italy (14.1%) or the Islands (5.9%). About half (51.8%) of the participants worked in facilities treating 50 to 500 patients hospitalized as a result of SARS-CoV-2 infection, followed by those working in facilities with fewer than 50 infected patients (30.3%). Less than 18% of the participants worked in facilities with more than 500 patients hospitalized for SARS-CoV-2 infection (11.2% with 501–1,500 patients, and 6.7% with more than 1,500 patients).

### Chest imaging modalities selection: phase 1 (Q6–Q9)

During the first phase of the COVID-19 pandemic, more than half of the participants (54.2%) predominantly performed chest CT scans to assess or exclude COVID-19 pneumonia (86.1% of whom used HRCT), followed by those who utilized CXR (42.6%). Only about 3% of the participants mainly relied on LUS in combination with CXR.

The choice to use chest CT during the first phase of the COVID-19 pandemic was significantly influenced by the geographical area in which the survey participants worked, with an increasing trend from Northern to Southern Italy and the Islands (*p* < 0.001) (Table 2). On the other hand, the amount of work experience, the workplace, and the number of patients hospitalized for SARS-CoV2 infection did not significantly affect the diagnostic tool selected to assess or exclude COVID-19 pneumonia (*p* ≥ 0.161) (Table 2).

**Table 2:** Chest imaging modalities during the first phase: sub-analysis for Q6

Question	Sub-group	Imaging modality		*p* value*
		CXR ± LUS	Chest CT	
Q6. During the phase 1 of the emergency, which diagnostic method did you most use to assess/exclude COVID-19 pneumonia?	Work experience (years)			
	< 10	72 (43.6)	93 (56.4)	0.601
	10—20	113 (46.5)	130 (53.5)	
	> 20	143 (46.4)	165 (53.6)	
	Workplace			
	University hospital	93 (47.0)	105 (53.0)	0.289
	Nonuniversity public hospital	176 (47.2)	197 (52.8)	
	Private facilities	59 (40.7)	86 (59.3)	
	Geographical area of work in Italy			
	North	204 (52.2)	187 (47.8)	< 0.001
	Centre	76 (41.8)	106 (58.2)	
	South and Islands	48 (33.6)	95 (66.4)	
	Patients hospitalized with infection			
	< 50	89 (41.0)	128 (59.0)	0.161
	50 – 500	178 (48.0)	193 (52.0)	
	> 500	61 (47.7)	67 (52.3)	

Data are presented as numbers (% of row total); CXR, chest X-ray; LUS, lung ultrasonography; CT, computed tomography; * *p*-values obtained by means the Cochran-Armitage test for trend

During the first phase of the COVID-19 pandemic, the least used imaging method for assessing or excluding COVID-19 pneumonia was LUS in combination with CXR (60.2% of the respondents). In such circumstances, LUS was mainly conducted by emergency physicians, followed by pulmonologists/internists, critical care physicians, and, finally, radiologists.

Based on the experience gained during the first phase, 312/716 (43.6%) respondents replied that they would have preferred to use a different imaging modality to assess/exclude COVID-19 pneumonia. Most participants (77.1%) would have preferred chest CT; of these respondents, 84% replied that they would have preferred HRCT. In particular, 199/305 (65.3%) participants who had used CXR responded that they would have preferred to perform chest CT. On the other hand, only 12.1% of respondents who had used chest CT stated that they would have preferred to perform CXR, either alone or in combination with LUS.

No relationship was found between the desire to change the diagnostic method (from CXR ± LUS to chest CT or vice versa) and the amount of work experience, the workplace, the geographical area in which the survey participants worked, or the number of patients hospitalized for SARS-CoV2 infection (p ≥ 0.124).

### Chest imaging modalities selection: phase 2 (Q10–Q12)

During the second phase of the COVID-19 pandemic, 65.6% of the participants had predominantly performed chest CT scans to assess or exclude COVID-19 pneumonia (86% of whom had used HRCT), followed by those who utilized CXR (32.4%). Only 14/716 (2%) participants predominantly used LUS in combination with CXR.

The decision to use chest CT during the second phase of the COVID-19 pandemic was significantly influenced by the workplace environment (more frequent in university hospitals), the geographical area in which the survey participants worked (more frequent in Southern Italy and the Islands), and the number of patients hospitalized for SARS-CoV2 infection (more frequent in facilities treating more than 500 patients) (*p* ≤ 0.045) (Table 3). On the other hand, the amount of work experience did not significantly affect the choice of the diagnostic tool used during the second phase (*p* = 0.931) (Table 3).

**Table 3:** Chest imaging modalities during the second phase: sub-analysis for Q10

Question	Sub-group	Imaging modality		*p* value*
		CXR ± LUS	Chest CT	
Q10. Since the beginning of the phase 2, which diagnostic method did you most use to assess/exclude COVID-19 pneumonia?	Work experience (years)			
	< 10	55 (33.3)	110 (66.7)	0.931
	10—20	86 (35.4)	157 (64.6)	
	> 20	105 (34.1)	203 (65.9)	
	Workplace			
	University hospital	51 (25.8)	147 (74.2)	0.037
	Nonuniversity public hospital	144 (38.6)	229 (61.4)	
	Private facilities	51 (35.2)	94 (64.8)	
	Geographical area of work in Italy			
	North	148 (37.9)	243 (62.1)	0.023
	Centre	58 (31.9)	124 (68.1)	
	South and Islands	40 (28.0)	103 (72.0)	
	Patients hospitalized with infection			
	< 50	82 (37.8)	135 (62.2)	0.045
	50 – 500	130 (35.0)	241 (65.0)	
	> 500	34 (26.6)	94 (73.4)	

Data are presented as numbers (% of row total); CXR, chest X-ray; LUS, lung ultrasonography; CT, computed tomography; * p-values obtained by means the Cochran-Armitage test for trend

During the second phase, the least used method of assessing or excluding COVID-19 pneumonia was LUS in combination with CXR (57% of the respondents).

During the second phase of the pandemic, 273/716 (38.1%) respondents stated that they would have preferred to use a different method to assess or exclude COVID-19 pneumonia, with most participants (71.3%) stating that they would have preferred to use chest CT. In particular, 131/232 (56.4%) participants who had used CXR stated that they would have instead preferred to use chest CT. On the other hand, about 20% of the respondents who had used chest CT (both HRCT and conventional CT) indicated that they would have preferred to use CXR alone or in combination with LUS.

The desire to change the diagnostic method (from CXR ± LUS to chest CT or vice versa) for evaluating/excluding COVID-19 pneumonia was significantly influenced by the workplace, with an increasing trend observed from university hospitals to private facilities (*p* = 0.027). No relationship was found between the desire to change the diagnostic method (from CXR ± LUS to chest CT or vice versa) for evaluating/excluding COVID-19 pneumonia and the amount of work experience, the geographical area in which the survey participants worked, or the number of patients hospitalized for SARS-CoV2 infection (*p* ≥ 0.121).

### Chest imaging modalities selection: monitoring (Q13, Q14)

During the first wave of COVID-19 (including both the first and second phases), 371/716 (51.8%) participants predominantly performed chest CT scans to monitor COVID-19 pneumonia (83% of whom used HRCT), followed by those who predominantly utilized CXR (45.3%). Only about 3% of the participants stated that they had mainly used LUS in combination with CXR.

The selection of chest CT for monitoring COVID-19 pneumonia was significantly influenced by the geographical area of work (with an increasing trend from Northern to Southern Italy and the Islands) and the number of patients hospitalized for SARS-CoV-2 infection (more frequent in facilities with fewer than 50 patients) (*p* < 0.001) (Table 4). On the other hand, neither the amount of work experience nor the workplace significantly affected the diagnostic imaging tool selected for the monitoring of COVID-19 pneumonia (p ≥ 0.358) (Table 4).

**Table 4:** Chest imaging modalities for monitoring COVID-19 pneumonia: sub-analysis for Q13

Question	Sub-group	Imaging modality		*p* value*
		CXR ± LUS	Chest CT	
Q13. During the COVID-19 emergency (both phase 1 and 2), which diagnostic method did you most use for monitoring COVID-19 pneumonia?	Work experience (years)			
	< 10	78 (47.3)	87 (52.7)	0.563
	10–20	114 (46.9)	129 (53.1)	
	> 20	153 (49.7)	155 (50.3)	
	Workplace			
	University hospital	99 (50.0)	99 (50.0)	0.358
	Nonuniversity public hospital	181 (48.5)	192 (51.5)	
	Private facilities	65 (44.8)	80 (55.2)	
	Geographical area of work in Italy			
	North	236 (60.4)	155 (39.6)	< 0.001
	Center	79 (43.4)	103 (56.6)	
	South and Islands	30 (21.0)	113 (79.0)	
	Patients hospitalized with infection			
	< 50	76 (35.0)	141 (65.0)	< 0.001
	50 – 500	201 (54.2)	170 (45.8)	
	> 500	68 (53.1)	60 (46.9)	

Data are presented as numbers (% of row total); CXR, chest X-ray; LUS, lung ultrasonography; CT, computed tomography; * p values obtained by means the Cochran-Armitage test for trend

Based on the experience gained during the first wave, 224/716 (31.3) respondents replied that would have used a different imaging method to monitor COVID-19 pneumonia. Most participants (58.8%) stated that they would have preferred to perform chest CT. In particular, 109/324 (33.6%) participants who had used CXR would have preferred to perform chest CT. On the other hand, about 18% of respondents who had used chest CT stated that they would have preferred to use CXR, either alone or in combination with LUS.

The desire to change the diagnostic method (from CXR ± LUS to chest CT or vice versa) used for the monitoring of COVID-19 pneumonia was significantly influenced by the number of patients hospitalized for SARS-CoV2 infection (with a decreasing trend from facilities treating fewer than 50 patients to hospitals treating more than 500 patients) (*p* = 0.021). At the limits of statistical significance, the influence of the radiologist’s workplace with an increasing trend from university hospitals to private facilities (*p* = 0.054). No relationship was found between the desire to change the diagnostic method and the amount of work experience or the geographical area of work (*p* ≥ 0.280).

### Reporting systems for COVID-19 pneumonia (Q15, Q16)

With regard to the reporting systems used for COVID-19 pneumonia in both phases, 398/716 (55.6%) participants stated that they had solely used a narrative reporting system, whereas 234/716 (32.7%) respondents stated that their narrative report also included a visual score. About 8% of the participants utilized structured reports, and only 3% of the respondents employed some type of software to quantify the extent of lung abnormalities.

The choice to use an unstructured narrative report was significantly influenced by the number of patients hospitalized for SARS-CoV2 infection, with a decreasing trend from facilities with fewer than 50 patients to hospitals with more than 500 patients (*p* < 0.001) (Table 5). On the other hand, the work experience, the workplace, and the geographical area of work did not significantly affect the choice to use narrative report for cases of COVID-19 pneumonia (*p* ≥ 0.090) (Table 5).

**Table 5:** Reporting systems for patients with COVID-19 pneumonia: sub-analysis for Q15

Question	Sub-group	Reporting systems		*p* value*
		Narrative	Other formats	
Q15. During the COVID-19 emergency (both phase 1 and 2), which reporting format did you use in patients with COVID-19 pneumonia?	Work experience (years)			
	< 10	86 (52.1)	79 (47.9)	0.599
	10–20	141 (58.0)	102 (42.0)	
	> 20	171 (55.5)	137 (44.5)	
	Workplace			
	University hospital	103 (52.0)	95 (48.0)	0.173
	Nonuniversity public hospital	209 (56.0)	164 (44.0)	
	Private facilities	86 (59.3)	59 (40.7)	
	Geographical area of work in Italy			
	North	204 (52.2)	187 (47.8)	0.090
	Center	110 (60.4)	72 (39.6)	
	South and Islands	84 (58.7)	59 (41.3)	
	Patients hospitalized with infection			
	< 50	148 (68.2)	69 (31.8)	< 0.001
	50 – 500	209 (56.3)	162 (43.7)	
	> 500	41 (32.0)	87 (68.0)	

Data are presented as numbers (% of row total); * p values obtained by means the Cochran-Armitage test for trend

Based on the experience gained during the first wave, 430/716 (60.1%) respondents stated that they would have used a different reporting system in COVID-19 pneumonia. In particular, most participants (68.6%) who had used narrative reports stated that they would have preferred to use other reporting systems (approximately 40% of whom would have preferred structured reports). In total, 261/716 (36.5%) participants indicated that they would have preferred to utilize a structured report; this percentage of respondents was four times higher than the percentage of those who actually used this reporting format (36.5% versus 8.7%, respectively).

The desire to use a structured report was significantly influenced by the geographical area of work, with a decreasing trend from Northern to Southern Italy and the Islands (*p* = 0.049) (Table 6). Just outside the limits of statistical significance the influence of the radiologists’ workplace on the desire to use structured reports for COVID-19 pneumonia, with a decreasing trend from university hospitals to private facilities (*p* = 0.074) (Table 6). No relationship was observed between the desire to use a structured report and the work experience or the number of patients hospitalized for SARS-CoV2 infection (*p* ≥ 0.576) (Table 6).

**Table 6:** Changing reporting format to structured report for COVID-19 pneumonia: sub-analysis of 654 participants who used other formats

Sub-group	Changing to structured report		*p* value*
	No	Yes	
Work experience (years)			
< 10	110 (70.1)	47 (29.9)	0.576
10—20	152 (69.4)	67 (30.6)	
> 20	188 (67.6)	90 (32.4)	
Workplace			
University hospital	118 (66.3)	60 (33.7)	0.074
Nonuniversity public hospital	229 (67.2)	112 (32.8)	
Private facilities	103 (76.3)	32 (23.7)	
Geographical area of work in Italy			
North	259 (71.5)	103 (28.5)	0.049
Center	114 (67.9)	54 (32.1)	
South and Islands	77 (62.1)	47 (37.9)	
Patients hospitalized with infection			
< 50	144 (71.6)	57 (28.4)	0.636
50 – 500	225 (66.6)	113 (33.4)	
> 500	81 (70.4)	34 (29.6)	

Data are presented as numbers (% of row total); * p values obtained by means the Cochran-Armitage test for trend

### Chest CT: image reconstruction and visualization (Q17–Q19)

With regard to the technical aspects used in chest CT examinations, most of the participants (approximately 85%) applied a protocol with thin-section CT images (reconstructed with a slice thickness of 1.5 mm or less) and high-spatial-frequency algorithms. While most participants (60.5%) used filtered back-projection (FBP) algorithms, just over a third (39.5%) of the respondents indicated that they applied iterative algorithms.

No relationship was found between the slice thickness (≤ 1.5 mm versus > 1.5 mm) and the amount of work experience, the workplace, the geographical area of work, or the number of patients hospitalized for SARS-CoV2 infection (*p* ≥ 0.137).

The use of iterative algorithms was significantly influenced by the geographical area of work (with a decreasing trend from Northern to Southern Italy and the Islands) and the number of patients hospitalized for SARS-CoV2 infection (with an increasing trend from facilities with fewer than 50 patients to hospitals with more than 500 patients) (*p* ≤ 0.018). No relationship was found between the use of iterative algorithms and the work experience or the workplace (*p* ≥ 0.265).

To assess the characteristics, extent, and distribution of lung abnormalities in cases of COVID-19 pneumonia, most of the participants (76.1%) indicated that they had utilized both cross sectional and multiplanar reformation (MPR) images in the coronal and sagittal planes, whereas approximately 15% of the respondents had used only cross-sectional images and only about 8% reported using an interactive three-dimensional (3D) CT viewer (6.4%) or advanced 3D analysis software (1.7%). No relationship was found between the use of MPR images and the amount of work experience, the workplace environment, the geographical area of work, or the number of patients hospitalized for SARS-CoV2 infection (*p* ≥ 0.090).

### Chest CT: low-dose, contrast media, and dual-energy (Q20–Q23)

During the first phase of the COVID-19 pandemic, 433/716 (60.5%) participants performed chest CT scans with low-dose protocols (of which 55.9% only in young patients, 31.4% in non-obese patients, and 12.7% always).

During the second phase of the COVID-19 pandemic, 471/716 (65.8%) respondents reported performing chest CT scans with low-dose protocols (of which 51.6% only in young patients, 35.4% in non-obese patients, and 13.0% always). The use of low-dose chest CT protocols during both phases was significantly influenced by the amount of work experience (with an increasing trend as the radiological experience increased) (*p* < 0.001). No relationship was found between the use of low-dose CT protocols and the workplace environment, the geographical area of work, or the number of patients hospitalized for SARS-CoV2 infection (*p* ≥  = 0.668).

With regard to intravenous contrast injection, 552/716 (77.1%) participants reported to administer contrast medium in patients with COVID-19 pneumonia, approximately 89% of whom had performed contrast-enhanced chest CT only in cases with suspected pulmonary embolism. The choice to use intravenous contrast material was significantly influenced by the workplace environment (with a decreasing trend from university hospitals to private facilities), the geographical area of work (with an increasing trend from Northern to Southern Italy and the Islands), and the number of patients hospitalized for SARS-CoV2 infection (with an increasing trend from facilities with fewer than 50 patients to hospitals with more than 500 patients) (*p* < 0.001).

With regard to the pulmonary angiography technique used in CT examinations, only 136/716 (19%) participants reported the availability of a dual-energy CT scanner; of these, only 25/136 (18.4%) respondents worked in facilities that had a dedicated protocol involving iodine mapping.

### Staff meetings, congresses, or courses (Q24–Q26)

With regard to staff meetings occurring in radiology departments during the COVID-19 pandemic (both phases 1 and 2), less than 15% of participants stated that staff meetings were organized regularly (weekly) in their facility, whereas about a third of participants said that no staff meetings occurred. The frequency of weekly staff meetings, which was reduced during the second phase, was significantly influenced by the workplace environment (higher in university hospitals than in other facilities) and the number of patients hospitalized for SARS-CoV2 infection (higher in hospitals with more than 500 patients) (*p* ≤ 0.022).

Finally, the responses to the last question indicated that almost all of the participants (94%) were interested in attending conferences or taking courses dedicated to the imaging of pulmonary infections.

## Discussion

The current pandemic has had, and continues to have, an impact on the life and work of all physicians, including radiologists. Several surveys have been conducted to explore various aspects of the impact of COVID-19 on professional activities (including educational and research activities) and the psychological well-being of radiologists [27–36], three of which have involved Italian radiologists [32–34].

To the best of our knowledge, this is the first Italian survey whose main objective was to investigate the factors influencing the selection of chest imaging modalities in the early phases of the pandemic and to assess, in hindsight, which radiological technique would have been preferred during the first wave of COVID-19 in Italy. Another relevant objective of this survey was to evaluate which reporting systems had been used to describe lung abnormalities in patients with COVID-19 pneumonia and which system would have been preferred by Italian radiologists.

Some of the results of this survey were unexpected, particularly concerning the most often used and preferred chest imaging modality for the diagnosis and follow-up of patients with COVID-19 pneumonia.

**Fig. 1:**
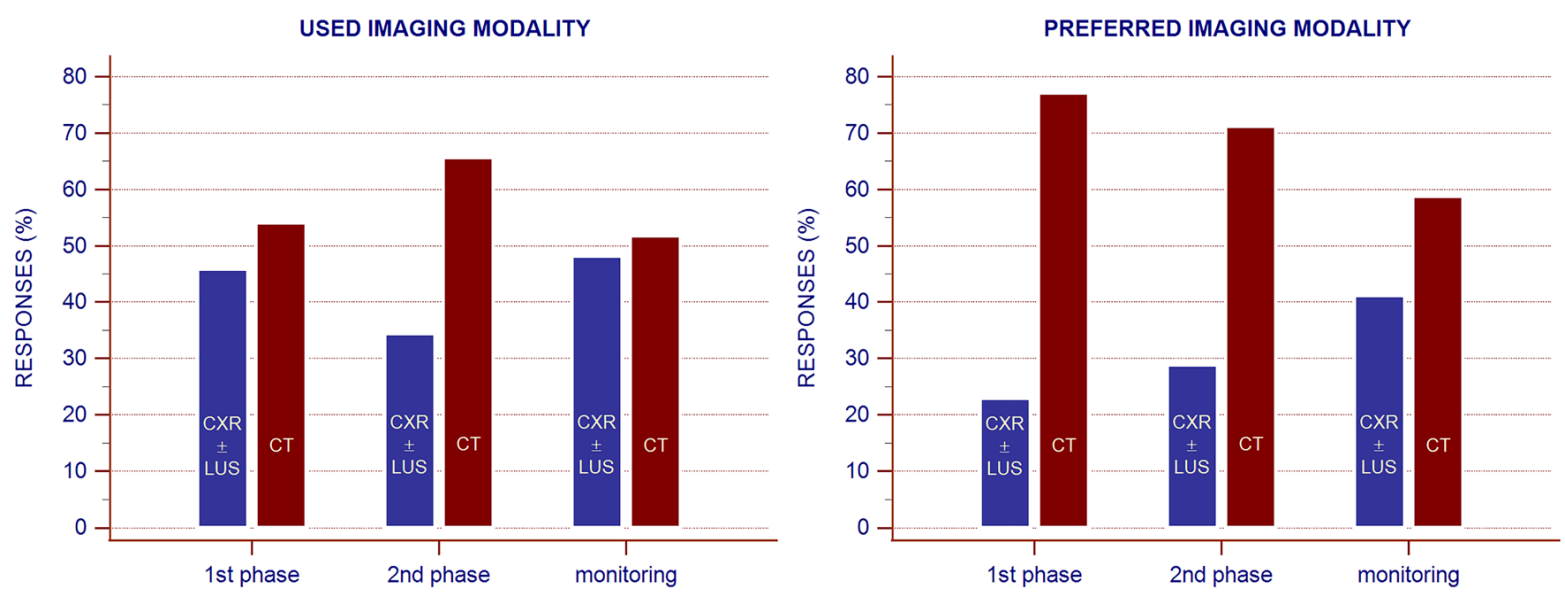
Bar chart showing the participants’ responses (%) on the chest imaging modalities used (*left*) and preferred (*right*) during the different phases of the first COVID-19 wave in Italy. CXR, chest X-ray; LUS, lung ultrasonography; CT, computed tomography

In contrast to the recommendations of several scientific societies, including those by the SIRM, which discourages the routine use of chest CT in patients with known or suspected COVID-19 pneumonia [15, 25, 37], we found that most survey participants (ranging from 51.8% to 77.1%) had used or would have preferred to use chest CT to assess/exclude/monitor COVID-19 pneumonia, regardless of the phases and pre-test probability (high, moderate, low) of infection (Fig. 1). The preference for chest CT was significantly related to the geographical area in which the survey participants worked, with an increasing trend from Northern to Southern Italy and the Islands. The highest frequency of CT use was observed in Southern Italy and the Islands, which could probably be explained by the fact that there were fewer infected patients in these geographical areas than in other regions, especially relative to those in Northern Italy, which experienced the highest rate of SARS-CoV-2 infection.

An interesting result was the way the number of hospitalized patients with COVID-19 impacted the decision to use CT imaging. While the frequency with which chest CT scan was used in the second phase was directly proportional to the number of patients hospitalized for COVID-19, the frequency of chest CT utilized for monitoring was inversely related to the number of hospitalized patients. This apparent contradiction may be explained by the fact that hospitals that had the highest number of admissions for COVID-19 in the first phase had (in a period of low disease prevalence) a greater need to quickly decide whether a patient should be discharged or hospitalized to reduce the pressure on the COVID-19 wards. Significant differences in the choice to using chest CT during the second phase were also observed in the sub-group analysis related to the workplace environment; particularly, university hospitals performed chest CT more frequently than other workplaces.

The least common method for assessing COVID-19 pneumonia was LUS in combination with CXR; this was not surprising, as radiologists perform LUS less frequently than other specialists, including emergency physicians, pulmonologists/internists, and critical care physicians. Additionally, the use of LUS in combination with CXR was also the least preferred imaging modality to assess/exclude/monitor cases of COVID-19 pneumonia in both phases of the first wave, probably due to the fact that the utility of ultrasound techniques greatly depends on the level of expertise of the operator [22–24].

Another interesting finding was that more than half of the respondents who had predominantly used CXR indicated that they would have preferred to use chest CT to assess/exclude COVID-19 pneumonia. The desire to change the diagnostic method from CXR to chest CT is likely due to the relatively higher sensitivity of chest CT in the detection of lung abnormalities (especially in the early stage of the disease) [13–20], as well as its superior ability to differentiate COVID-19 pneumonia from other infectious and noninfectious interstitial diseases [38–40]. However, CXR does have several advantages in the management of patients with suspected or confirmed COVID-19 infection [6–13]. Although some radiologists and clinicians consider the information provided by CXR to be insufficient, a recent study demonstrated that the use of CXR instead of chest CT as a first-line imaging modality in a simulated COVID-19 pandemic triage was safe and helped optimize both the use of radiology resources and patient management [14]. Additionally, considering the rapid progression of lung abnormalities that occurs in cases of COVID-19 pneumonia, we believe that CXR, either alone or in combination with LUS, is the most appropriate diagnostic imaging tool for day-to-day monitoring of the course of the disease, particularly in critically ill patients.

With regard to the reporting systems used for COVID-19 pneumonia, several scientific societies have recommended the use of a standardized reporting scheme and language for chest CT to improve communication between radiologists and clinicians [37, 41, 42]. In particular, the SIRM has conceived and proposed a helpful structured reporting scheme for chest CT evaluations that can be utilized by all radiologists, regardless of their experience in chest imaging [37]. In the present study, we observed that more than half of the participants used an unstructured narrative report, and this reporting format was used significantly less frequently among respondents who worked in hospitals with more than 500 patients with COVID-19. This finding is likely due to the fact that larger hospitals became overwhelmed by a progressive increase in demand for radiological examinations, and this increased workload favored the introduction of other types of reporting, such as those involving a scoring system or structured reports. Additionally, most participants who used narrative reports indicated that they would have preferred to use other reporting systems, with about 40% preferring structured reports. The desire for structured reporting systems was expressed more frequently among participants working in the Southern Italy and Islands, probably due to the fact that in those geographical areas chest CT is the most commonly used and most preferred imaging modality to assess and monitor cases of COVID-19 pneumonia.

As for the chest CT acquisition and reconstruction technique used, most of the participants had applied a high-resolution protocol, which is the most accurate to assessing diffuse lung diseases. Approximately a third of the respondents reported using iterative algorithms, a reconstruction technique that was used more frequently in those working in Northern Italy and in hospitals with more than 500 patients with COVID-19. This finding was probably due to the increased availability of CT scanners with iterative algorithms in Northern Italy and in larger hospitals.

We greatly appreciated that most of the participants had used both cross-sectional and MPR images to assess the characteristics, extent, and distribution of lung abnormalities in COVID-19 pneumonia. Additionally, we were surprised to find that over 60% of the participants had used low-dose protocols to perform chest CT scans to assess COVID-19 pneumonia, a strategy that was employed more frequently among the more experienced radiologists.

With regard to intravenous contrast injection in patients with confirmed or suspected COVID-19 pneumonia, most of the participants reported to perform contrast-enhanced CT scans only in cases in which a pulmonary embolism was suspected. As expected, the use of contrast media occurred significantly more frequently in large hospitals and in geographical areas experiencing a high number of admissions for COVID-19. Unfortunately, however, only a small percentage of the participants working in facilities with an available dual-energy CT scanner had a dedicated protocol involving iodine mapping for patients with a suspected pulmonary embolism.

Also problematic was the fact that less than 15% of the survey participants stated that staff meetings were organized regularly (weekly) during the first wave of COVID-19, and about a third reported that such meetings were never organized. Unsurprisingly, weekly staff meetings occurred significantly more frequently in university hospitals and in facilities with more than 500 patients with COVID-19.

The main limitations of this study include the low survey response rate (corresponding to 6.8% of all active members of the SIRM) and the disproportionately higher rate of participation by radiologists working in Northern Italy (corresponding to 54.6% of all survey participants). However, the in-depth analysis of the survey responses, which comprised sub-group analyses based on various baseline characteristics of the participants, should have counterbalanced these limitations.

In conclusion, this study provides a comprehensive overview of the chest imaging modalities that have been employed by Italian radiologists for the diagnosis and follow-up of patients with COVID-19 pneumonia, as well as the factors that have contributed to the selection of specific methods in different contexts and in different period of time during the first wave of the pandemic. As the participants’ responses did not properly align with the recommendations that have been made by several scientific societies, including the SIRM, we believe there is a persistent need for continuing education to keep radiologists up to date and aware of the advantages and limitations of the chest imaging modalities and reporting systems used in the management of patients with pulmonary infections, including potentially severe forms such as COVID-19.
